# Systematic Identification of Suitable Reference Genes for Quantitative Real-Time PCR Analysis in *Melissa officinalis* L

**DOI:** 10.3390/plants12030470

**Published:** 2023-01-19

**Authors:** Rohit Bharati, Madhab Kumar Sen, Ram Kumar, Aayushi Gupta, Jana Žiarovská, Eloy Fernández-Cusimamani, Olga Leuner

**Affiliations:** 1 Department of Crop Sciences and Agroforestry, The Faculty of Tropical AgriSciences, Czech University of Life Sciences Prague, Kamýcká 129, 165 00 Prague, Czech Republic; 2 Department of Agroecology and Crop Production, Faculty of Agrobiology, Food and Natural Resources, Czech University of Life Sciences Prague, Kamýcká 129, 165 00 Prague, Czech Republic; 3 Department of Plant Protection, Faculty of Agrobiology, Food and Natural Resources, Czech University of Life Sciences Prague, Kamýcká 129, 165 00 Prague, Czech Republic; 4 Department of Botany and Plant Physiology, Faculty of Agrobiology, Food and Natural Resources, Czech University of Life Sciences Prague, Kamýcká 129, 165 00 Prague, Czech Republic; 5 Research Centre AgroBioTech, Slovak University of Agriculture in Nitra, Tr. A. Hlinku 2, 94976 Nitra, Slovakia

**Keywords:** abiotic stress, elicitor treatment, gene expression, qRT-PCR, lemon balm, reference genes

## Abstract

*Melissa officinalis* L. is well known for its lemon-scented aroma and various pharmacological properties. Despite these valuable properties, the genes involved in the biosynthetic pathways in *M. officinalis* are not yet well-explored when compared to other members of the mint family. For that, gene expression studies using quantitative real-time PCR (qRT-PCR) are an excellent tool. Although qRT-PCR can provide accurate results, its accuracy is highly reliant on the expression and stability of the reference gene used for normalization. Hence, selecting a suitable experiment-specific reference gene is very crucial to obtain accurate results. However, to date, there are no reports for experiment-specific reference genes in *M. officinalis*. Therefore, in the current study, ten commonly used reference genes were assessed for their suitability as optimal reference genes in *M. officinalis* under various abiotic stress conditions and different plant organs. The candidate genes were ranked based on BestKeeper, comparative ΔCt, geNorm, NormFinder, and RefFinder. Based on the results, we recommend the combination of *EF-1α* and *GAPDH* as the best reference genes to normalize gene expression studies in *M. officinalis*. On the contrary, *HLH71* was identified as the least-performing gene. Thereafter, the reliability of the optimal gene combination was assessed by evaluating the relative gene expression of the *phenylalanine ammonia lyase* (*PAL*) gene under two elicitor treatments (gibberellic acid and jasmonic acid). *PAL* is a crucial gene involved directly or indirectly in the production of various economically important secondary metabolites in plants. Suitable reference genes for each experimental condition are also discussed. The findings of the current study form a basis for current and future gene expression studies in *M. officinalis* and other related species.

## 1. Introduction

*Melissa officinalis* L., commonly known as the lemon balm is a medicinal herb belonging to the mint family (Lamiaceae). It is well known for its characteristic lemon-scented aroma. It has applications in several industries such as agriculture, sanitary, food, and beverages [1,2]. Traditionally, it has been used to remedy gastrointestinal disorders and several other conditions such as sore throat, cough, insomnia, vertigo, nausea, rheumatoid arthritis, and neurological disorders [1,2]. Additionally, several recent studies have also demonstrated *M. officinalis* to possess hypolipidemic, antioxidant, antimicrobial, anxiolytic, anticancer, and anti-inflammatory effects [2,3,4]. These pharmacological properties of medicinal and aromatic plants are mainly attributed to the secondary metabolites and essential oils present in the plant [1,5]. Several successful attempts have been made by researchers to increase these metabolites through bio-stimulants, nanoparticles, hormones, and various elicitor treatments. For instance, up to a 7.43-fold increase in rosmarinic acid was observed in *M. officinalis* when treated with methyl jasmonate [6]. Moreover, studies have also employed numerous in vitro approaches such as callus culture, cell suspension culture, and bioreactors to enhance secondary metabolite production in medicinal and aromatic plants [7,8,9,10]. These attempts will further increase to fulfill the rising global demand for these secondary metabolites and essential oils across industries. To achieve this, a better understanding of the crucial biosynthetic pathways involved is a prerequisite, mainly ones associated with secondary metabolites. Genes involved in these biosynthetic pathways are well studied across the mint family; however, very little has been explored in *M. officinalis* when compared to other members of the mint family. Although, a recent transcriptome analysis attempted to identify various key genes involved in the biosynthesis of terpenoids and phenylpropanoids [11]. For further understanding of other crucial biosynthetic pathways and genes involved in those pathways of this plant species, gene expression studies appear as an effective tool.

Quantitative real-time PCR (qRT-PCR) is a sensitive, rapid, and efficient approach for comparative expression studies. Despite qRT-PCR being able to provide accurate results, its accuracy is highly dependent on the expression and stability of the housekeeping gene used to normalize the qRT-PCR data [12,13,14,15]. Transcription levels of the target gene must be normalized with the transcription level of a suitable reference gene having stable expression across all treatments and conditions [16]. Some of the commonly utilized reference genes for gene expression studies in plants include *glyceralde-hyde-3-phosphate dehydrogenase* (*GAPDH*), *α-tubulin* (*α-TUB*), *β-tubulin* (*β-TUB*), *actin* (*ACT*), *ubiquitin* (*UBQ*), *ribosomal RNA* genes (rRNA), *Acetyl CoA Carboxylase* (*ACCase*), *eukaryotic elongation factor* (*eEF*), etc. [12,16]. Although it can be assumed that these genes will have stable expression levels across any given condition, several studies have demonstrated that the expression of these genes could vary considerably. Sometimes these variations can be found across different plant species, development stages, or even plant organs [17,18,19]. Hence, any failures while choosing the suitable reference gene/s might be misleading.

To date, a systematic study for the evaluation or validation of reference genes for qRT-PCR analysis in *M. officinalis* has not been reported. Hence, the current study aims to screen out the most suitable reference gene/s for *M. officinalis* among ten of the commonly utilized reference genes in plant gene expression studies: *ATP-synthase*, *F1-ATPase*, *60S rRNA*, *28S rRNA*, *HLH71, β-tubulin*, *25S rRNA*, *glutathione S-transferase* (*GST*), *GAPDH*, *elongation-factor-1-alpha* (*EF-1α*) and *Ubiquitin* [12,13,20]. The genes were screened with various plant organs (young leaves, mature leaves, and stems) and abiotic stress conditions (heat, cold, salt, osmotic, and in vitro conditions). Thereafter, five statistical algorithms for optimal reference gene analysis (BestKeeper [21], comparative ΔCt [22], geNorm [23], NormFinder [24], and RefFinder [25]) were used to screen the best candidate. To assess the reliability of the selected reference gene, the relative expression of *phenylalanine ammonia lyase* (*PAL*) was analyzed under two elicitor treatments jasmonic acid and gibberellic acid. *PAL* is a crucial enzyme that catalyzes the phenylpropanoid pathway, an important secondary metabolite pathway. This enzyme plays a major role in the production of several compounds such as anthocyanin, caffeic acid, flavonoid, lignin, and phenylpropanoids [6,26,27]. Along with the best candidate, suitable reference gene/s under each experimental condition were also identified. The findings of the current study form a basis for current and future gene expression studies in *M. officinalis* and other related species.

## 2. Results

### 2.1. Primer Efficiency and the Expression Profile of the Candidate Genes

**Figure 1:**
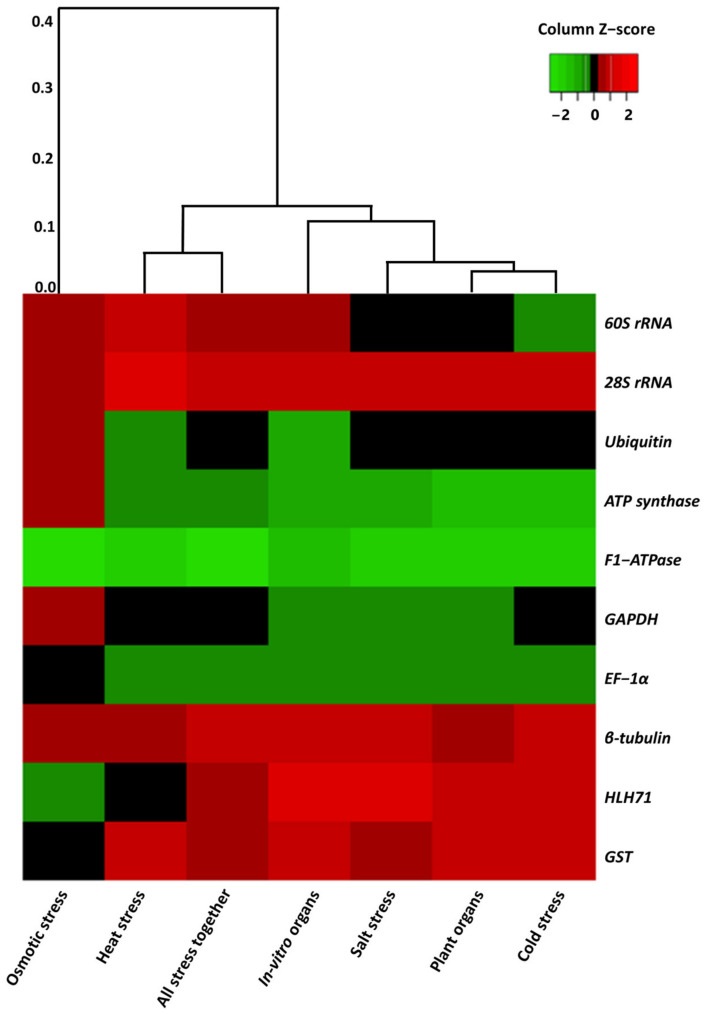
Heatmap of candidate reference genes based on their Ct or cycle threshold values. Expression levels of the candidate genes is represented by three main colors (green, black, and red). The red color indicates a high Ct value or low expression and the green color indicate a low Ct value, which corresponds to high expression. The black color represents neither high nor low Ct values (moderate values).

**Figure 2:**
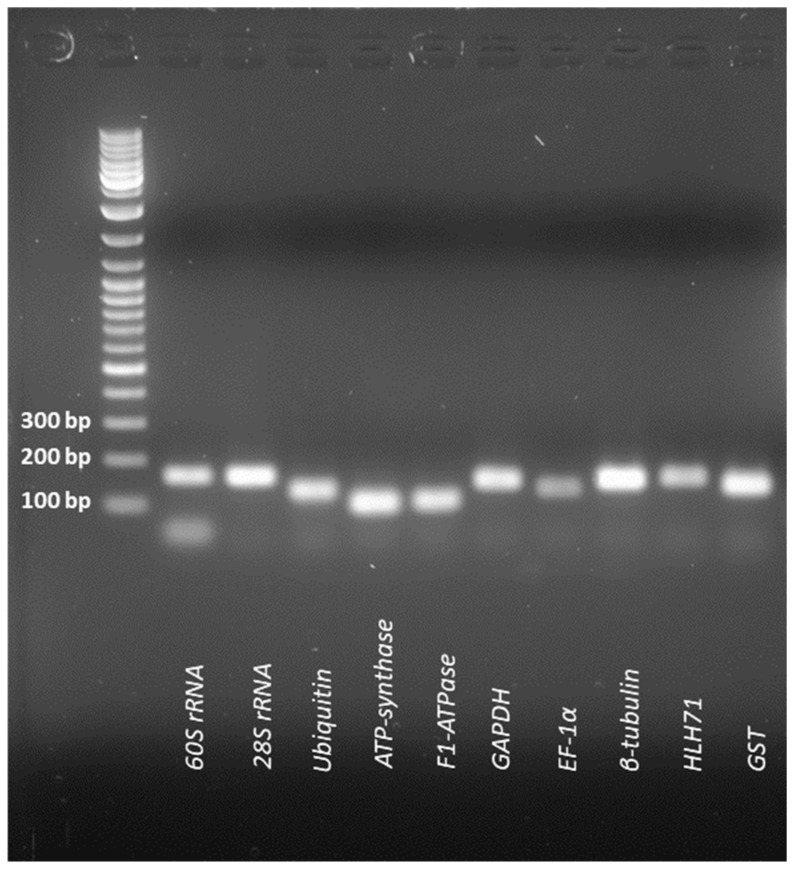
Candidate reference genes on 1.8% agarose gel with single bands for each.

**Figure 3:**
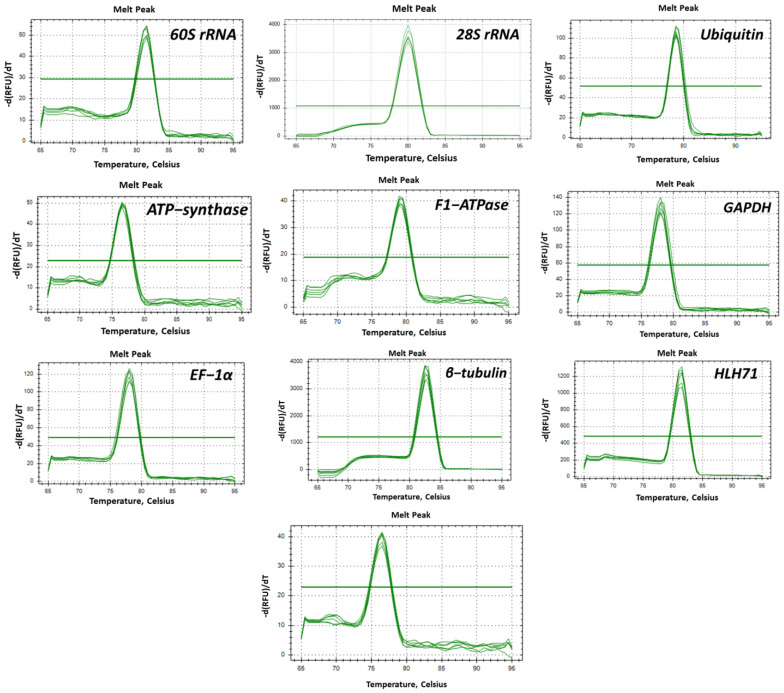
Melting curve of the candidate genes.

The primer efficiency values ranged from 97.33 to 104.86% and the correlation coefficient values ranged from 0.97 to 1.00, which are under the acceptable range (Table 1). The expression profiles and patterns of the 10 candidate genes under different experimental conditions are shown in Figure 1. In the case of all the designed primers, a single band (in 1.8% agarose gel) and a single peak (qRT-PCR amplification) were obtained (Figure 2 and Figure 3, respectively). The Ct values of the 10 nominees exhibited a wide-ranging value in all samples. Irrespective of the experimental conditions, *F1-ATPase* showed the lowest Ct value, indicating high expression of the gene.

### 2.2. Expression Stability of Candidate Reference Genes by Comparative ΔCt and Bestkeeper

The comparative ΔCt and BestKeeper were used to calculate the stability of the candidate reference genes based on the average standard deviation and the standard deviation (+/- crossing point) values, respectively. The expression stability of candidate genes analyzed by ΔCt and BestKeeper is shown in Table 2. According to the comparative ΔCt analysis, *GAPDH* can be considered the most stable reference gene for plant organs and salt stress. However, in these stresses, BestKeeper recommends using *GST*. In the cases of heat and osmotic stress, comparative ΔCt analysis is suggested using *28S rRNA* whereas according to the BestKeeper analysis, *HLH71* is the best candidate. For cold stress, comparative ΔCt and BestKeeper analyses recommend using *EF-1α* and *60S rRNA*, respectively. In the cases of all stress combined, *28S rRNA* and *GST* were indorsed by comparative ΔCt and BestKeeper analyses, respectively. For experiments with in vitro and all conditions combined, comparative ΔCt analysis proposes using *β-tubulin* and *28S rRNA*, correspondingly. According to BestKeeper analysis, for experiments under in vitro conditions and all conditions combined, *HLH71* and *F1-ATPase* (respectively) were highly recommended.

### 2.3. Expression Stability of Candidate Reference Genes by NormFinder

NormFinder software ranked the candidate genes based on their stability values. This software first assessed the intra- and inter-group variations, which are then combined into a direct variation value, which is also known as stability value. The gene with the least variation is finally ranked as the best by the software. The expression stability of candidate genes analyzed by NormFinder is shown in Table 3. According to the NormFinder analysis, *GAPDH* is the best reference gene for all the experimental conditions tested in this manuscript, except for experiments under osmotic stress and in vitro conditions. For osmotic stress, the software suggests using *28S rRNA* whereas under in vitro conditions, using *EF-1α* is highly recommended.

### 2.4. Expression Stability of Candidate Reference Genes by GeNorm

**Figure 4:**
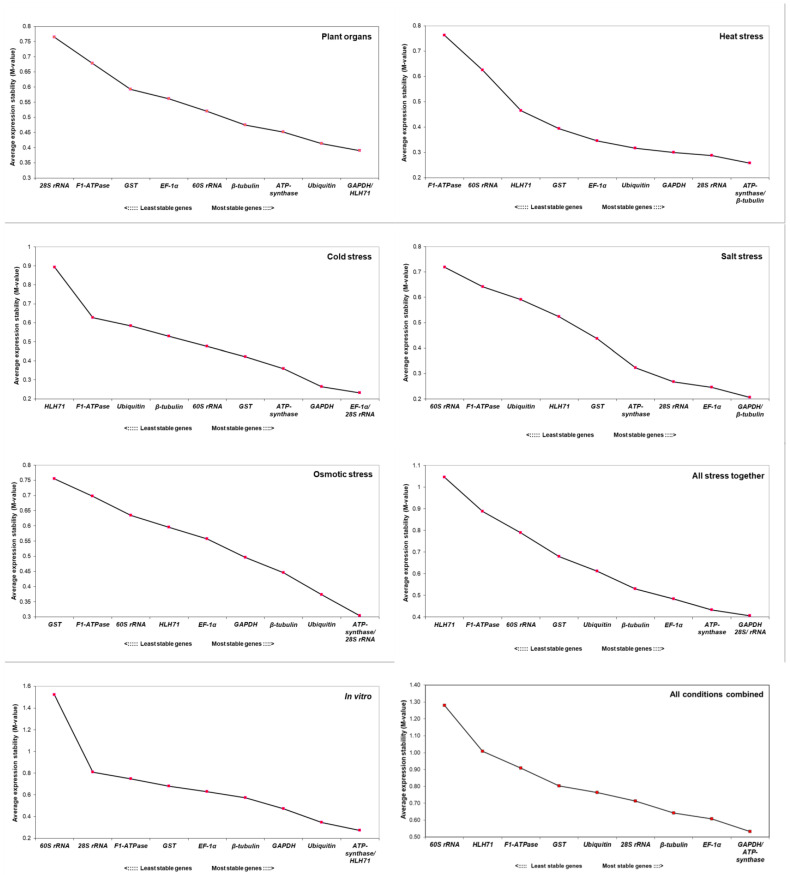
geNorm ranking of the candidate genes under different tested conditions.

**Figure 5:**
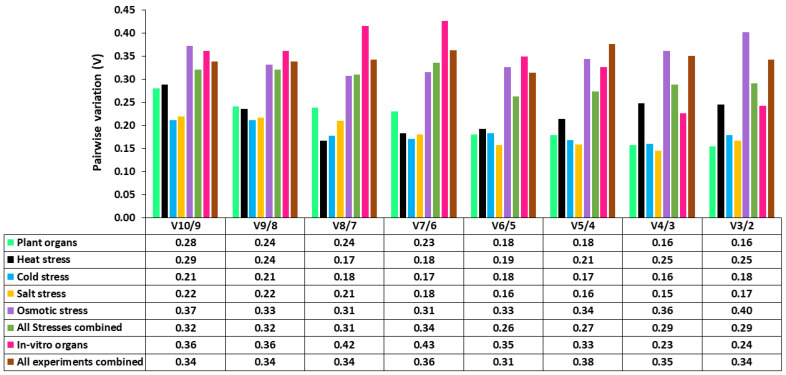
Pairwise variation to determine the optimal number of reference genes. The recommended cutoff value under which there is no need for another gene is 0.15.

In geNorm analysis, the software evaluates the gene expression values according to their respective M values. The findings of geNorm analyses are shown in Figure 4. According to our analysis, the most stable reference gene varied across different experiments. In the case of experiments with plant organs, geNorm recommends using a combination of *GAPDH* and *HLH71*, whereas, for experiments under heat stress, a combination of *ATP-synthase* and *β-tubulin* is recommended. In the case of cold, salt, and osmotic stress, geNorm analyses suggest using a combination of *EF-1α* and *28S rRNA*, *GAPDH* and *β-tubulin* and *ATP-synthase* and *28S rRNA*, respectively. When all stresses are considered, this software recommends using a combination of *GAPDH* and *28S rRNA*. For gene expression experiments with in vitro *M. officinalis* plants, geNorm analyses recommend using a combination of *ATP-synthase* and *HLH71*. When all experimental conditions are taken together, geNorm analyses using a combination of *GAPDH* and *ATP-synthase*. Additionally, we also identified the optimal number of genes required for each experimental condition. The cut-off M-value for the optimal number of required genes is 1.5. In the present study, the pairwise variation value of all the datasets (V_n_/V_n+1_) was found to be higher than the recommended value, i.e., 0.15 (Figure 5). Nevertheless, these values are only indicative, and using a higher number of reference genes would increase experimental complexity and instability, eventually leading to ambiguous results. Considering that the V_n_/V_n+1_ value is indicative and not a mandatory criterion, one or a combination of two reference genes could be adequate for the normalization of the qRT-PCR data in *M. officinalis*.

### 2.5. Comprehensive Ranking of the Candidate Reference Genes by RefFinder

RefFinder web-based tool combines geNorm, NormFinder, BestKeeper, and Ct values, and provides a comprehensive ranking. According to the RefFinder, *GAPDH* can be deemed as the most stable reference gene for experiments with plant organs and under salt stress. The expression stability of candidate genes analyzed by RefFinder is shown in Table 4. In the case of experiments under heat stress, osmotic stress, and all stresses combined, *28S rRNA* is the most stable reference gene. For the gene expression experiments under cold stress and for in vitro conditions, RefFinder endorses using *GST* and *β-tubulin*, respectively. While considering all the experiments together, RefFinder recommends using *28S rRNA*.

### 2.6. Identification of the Most Suitable Reference Gene and Validation Experiments

**Figure 6:**
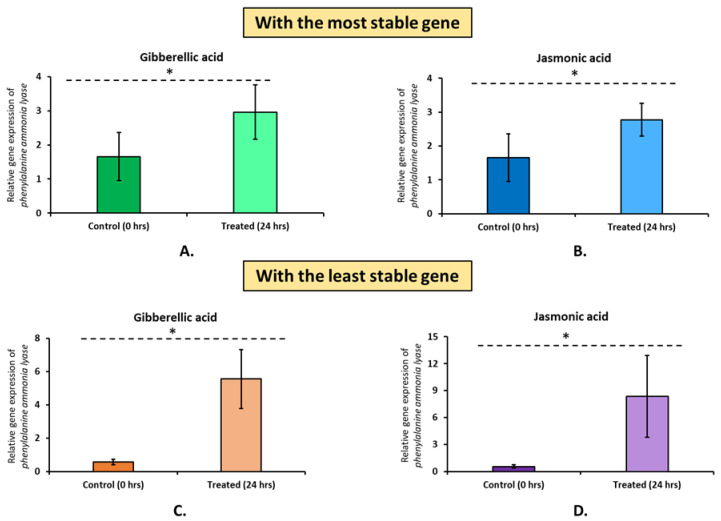
Relative expression level of *phenylalanine ammonia lyase (PAL)* under gibberellic acid stress (**A**,**C**) and jasmonic acid stress (**B**,**D**). Relative gene expression before elicitor treatment (Control) and after treatment (the most stable and the least stable gene) were compared, and normalization was performed with *EF-1α*|*GAPDH* (the most stable gene) and *HLH71* (the least stable gene). The relative gene expression between the control and elicitor-stressed samples was compared by Student’s *t*-test, “*” on the vertical bars indicating a significant difference at *p* < 0.05.

Based on comprehensive analysis by RefFinder, a combination of *EF-1α* and *GAPDH* was identified as the most appropriate reference gene for gene expression studies in *Melissa officinalis*. On the contrary, *HLH71* as a reference gene for gene expression studies in lemon balm is not recommended. Suitable genes for each experimental condition were also selected. For experiments under plant organs and heat stress, ranking suggests using a combination of *GAPDH* and *β-tubulin.* In the case of experiments under cold stress, our analysis recommends using a combination of *EF-1α* and *28S rRNA,* whereas, for experiments under salt stress, we endorse using a combination of *GAPDH* and *β-tubulin.* For experiments under osmotic stress and all stresses combined, we suggest using a combination of *ATP-synthase* and *28S rRNA* and *GAPDH* and *28S rRNA,* respectively. In the case of experiments with in vitro organs, we recommend using *EF-1α* and *β-tubulin.* Further, to substantiate the reliability of the candidate gene, the relative expression of the *PAL* gene under two different elicitor stresses was evaluated. Both the best and the least stable candidate genes were used for normalization. Irrespective of the reference gene type and elicitor type (gibberellic acid and jasmonic acid), we found elevated relative gene expression levels of *PAL* (Figure 6). In the case of both elicitors, when normalized with the best reference gene, the *PAL* gene shows ~2X overexpression compared to the untreated control. However, when normalized with *HLH71*, the *PAL* gene showed ~10X and ~15X overexpression under gibberellic acid and jasmonic acid stress, respectively.

## 3. Discussion

Gene expression studies using qRT-PCR have been used across numerous research studies to unravel the molecular mechanisms behind the biosynthetic pathways of various medicinal and aromatic plants [6,28,29,30,31]. With substantial progress, many genes and gene families have been identified that are directly or indirectly responsible for the synthesis of economically important secondary metabolites [31,32,33]. To identify these genes, selecting suitable reference genes for qRT-PCR analyses is crucial to avoid misleading results. The expression and stability of any given reference gene could vary greatly under different experimental conditions [17,18,19]. Consequently, several studies have identified experiment-specific reference genes in various plant species [18,19]. Despite this progress across numerous plant species, it is surprising that a suitable reference gene in *M. officinalis* has never been established, in any experimental condition. To date, gene expression studies in *M. officinalis* have mostly (virtually all the studies) utilized different random reference genes, presuming the selected gene has a stable expression. Hence, the current study is the only study to systematically identify the most suitable reference genes in lemon balm.

In this study, we assessed the stability of ten candidate genes under various experimental conditions to find the best reference gene in *M. officinalis*. For this purpose, five commonly used software and algorithms for reference gene selection were used. Based on the pairwise variation analysis, we found that a combination of two reference genes will be sufficient for normalization. The geNorm and the NormFinder are specialized software, which can predict the best combination based on expression stability values. According to geNorm, under all the combined stress conditions, the best pair is *GAPDH* and *ATP-synthase*, whereas, under similar conditions, NormFinder recommends using a combination of *EF-1α* and *GAPDH*. However, the comprehensive ranking software, RefFinder recommends not using *ATP-synthase* in lemon balm. Finally, based on our comprehensive analyses, a combination of *EF-1α* and *GAPDH* was found to be the best reference gene pair whereas *HLH71* was found to be the least-performing gene. *EF-1α* plays important roles during several important processes required for cell growth and proliferation, apart from its role in translation, elongation, and the nuclear export of proteins [34]. Similarly, *GAPDH* also plays important roles in several non-metabolic processes, such as transcription activation, apoptosis, and axoplasmic transport [35]. Hence, considering their important roles, these genes are expected to be ubiquitously expressed. Moreover, our results are consistent with a previous study where *EF-1α* and *GAPDH* along with *actin* were found to be the most suitable reference gene combination in two thyme species [36]. Similarly, *GAPDH* was also found to be a suitable reference gene in *Rosmarinus officinalis* L. and *Salvia hispanica* L. [31,37]. In *Isodon rubescens* (another member of the lamiaceae family), using a combination of *GAPDH*, *18S,* and *eIF* was recommended [38]. However, these results are not consistent across the lamiaceae and other families, for instance, *EF1* and *EF-1α* have been reported to be the least-performing genes in some members of the lamiaceae family [39,40]. In another study, a combination of *F1-ATPase*, *ATP synthase,* and *ACCase* was ranked as the best reference gene for gene expression studies in in vitro-produced rosemary [41]. For *Populus euphratica*, the optimal reference genes varied for different treatments (e.g., *RPL17* was selected for ABA and *EF-1α* was selected for cold) [42]. In blueberry, *GAPDH, ATP1, NADH,* and *COX2* genes showed high stability [43]. These studies evidently suggest that no reference gene can be considered a universal gene.

Further, the best and least gene was used to normalize the gene expression of the *PAL* gene under two elicitor treatments (gibberellic acid and jasmonic acid) to affirm its reliability. It is well established that plant hormone treatment such as gibberellic acid and jasmonic acid, stimulates secondary metabolites production by influencing the genes involved in phenylpropanoid pathway of the plant [6]. Correspondingly, in the current study, the expression of the *PAL* gene was elevated by the hormone treatments. However, in the case of normalization with the least stable gene, the expression levels of *PAL* were abnormally high, which clearly indicates the impact of using an unsuitable reference gene. Previously, a similar type of result was obtained in a study under herbicide stress by Sen et al. [12], where an herbicide target gene showed abnormal expression when treated with herbicide. Similarly, in our case, treatment with the elicitors is expected to increase the expression of the *PAL* gene, but in the case of normalization with the least stable reference genes, the expression pattern is misleading.

Overall, the reference genes recommended in this study can be used for gene expression studies under various developmental stages and diverse abiotic stresses in *Melissa officinalis* and other related species. We recommend using our reference genes to avoid any misleading results in qRT-PCR experiments with lemon balm and other related species.

## 4. Materials and Methods

### 4.1. Plant Material Acquisition, Stress, and Elicitor Treatment

Seed samples of *M. officinalis* were collected from the well-maintained botanical garden of the Faculty of Tropical AgriSciences (FTZ), Czech University of Life Sciences, Prague (50°07′52.9″ N, 14°22′14.7″ E). No prior specific permissions were necessary to collect the seed samples. The seeds were then sown in plastic pots (9 × 9 cm^2^) containing garden soil (Agro profi RS1, ‘s-Gravenzande, Netherlands) and perlite in a 3:1 ratio until germinated. Thereafter, the seedlings were maintained in greenhouse conditions with an average temperature of 25 °C and relative humidity between 60 and 70% for 30 days before subjecting to treatments. Five pots per treatment and one plant in each pot were maintained for 24 h. The treatments included: heat stress (35 °C), cold stress (4 °C), salt stress (NaCl, 200 mM), drought stress (PEG 6000, 200 mM), jasmonic acid (1 mg/L), gibberellic acid (1 mg/L) and control (plants without any treatment). The heat and cold stresses were carried out in a growth chamber (MLR-351H, Sanyo, Osaka, Japan). To induce the salt and drought stress, respective solutions were added directly to the pots whereas foliar treatments were used for elicitor treatments (jasmonic acid and gibberellic acid). The samples were collected and stored at −80 °C until further use. Additionally, samples were also collected of plant organs (young leaves, mature leaves, and stems) and in vitro plant organs. For in vitro plant organs, nodal segments were surface sterilized using a previously established protocol [44] and inoculated on MS basal media (without any phytohormones). After 30 days, in vitro plant organs (young leaves, mature leaves, and stems) were collected and stored at −80 °C until further use.

### 4.2. Total RNA Extraction, cDNA Synthesis, and qRT-PCR Experiment Conditions

Total RNA was extracted from the plant tissues (±60–80 mg per sample) using the RNeasy Mini Kit (Qiagen, Hilden, Germany). The purity and integrity of the RNA samples were assessed using a NanoDrop 2000 spectrophotometer (Thermo Scientific, Waltham, MA, USA) and running the samples on a 1.2% agarose gel electrophoresis. For cDNA synthesis, TURBO DNA-free™ (Invitrogen, Waltham, MA, USA) Kit was used according to the manufacturer’s instructions where 1μg (per sample) of the quality-checked gDNA-free RNA was used as the template. Ten commonly utilized reference genes in plant gene expression studies were selected and degenerated primers were designed (*ATP-synthase, F1-ATPase, 60S rRNA, 28S rRNA, HLH71, β-tubulin, GST, GAPDH, EF-1α,* and *Ubiquitin*). These genes were selected based on previous literature studies and the primers were designed based on the homologous sequence of related or other plant species using Primer3 software [(version 0.4.0) https://bioinfo.ut.ee/primer3-0.4.0/ (accessed on 15 August 2022)]. All primers designed were tested using a general PCR (C1000 thermocycler, Bio-Rad, Hercules, CA, USA) and confirmed on a 1.8% agarose gel electrophoresis. For the qRT-PCR analysis, CFX Connect Real-Time PCR Detection System (Bio-Rad Laboratories, Hercules, CA, USA) was used. The used reaction mixture was as follows: 5 μL of SYBR Green Master Mix (Applied Biosystems™, USA), 1 μL of primer mix (10 µM each), and 4 μL of cDNA (10 ng in total). The real-time thermocycler was programmed at an initial denaturation step at 95 °C for 10 min, followed by 40 cycles of 15 s at 95 °C and 1 min at 58.4 to 61 °C (based on the annealing temperature of the primer pairs). For the melting curves, stepwise heating was performed from 60 to 95 °C. To assess the reliability of the selected reference gene/s, the expression levels of *phenylalanine ammonia lyase (PAL)* were normalized using the best and the least stable gene under two elicitor treatments. The experiment was carried out with five biological and technical replicates.

### 4.3. Reference Gene Analysis

Gene expression stabilities of the candidate genes were examined by NormFinder [24], RefFinder (https://heartcure.com.au) (accessed on 15 August 2022) [25], comparative ΔCt [22], BestKeeper [21], and geNorm [23] according to a previous study [12]. The NormFinder software relies on the stability values and the variation among them to rank the genes, and genes with the least variations are ranked as the best. The geNorm software package utilizes the expression stability value (M) to rank the candidate genes where the M value is inversely proportional to the gene ranking. BestKeeper relies on the standard deviation values of crossing point values (CP) or cycle threshold (Ct) and the coefficient of correlation (*r*) values. The RefFinder software integrates the ranking from other software to offer a complete ranking based on the geometric means of the ranking value.

### 4.4. Statistical Analysis

The relative expression of the *PAL* gene between the untreated control and the treated samples was compared using a two-sample *t*-test in Microsoft Excel 2021 with XLSTAT (a Statistical Software for Excel) (version 2022.1) (https://www.xlstat.com/en/) (accessed on 27 August 2022). The average Ct values from five biological replicates were used for the calculation of the relative *PAL* gene expression level. The gene expression levels were calculated using the 2^−∆∆Ct^ method [22].

## 5. Conclusions

In summary, the current study is the first systematic study to identify and validate the most suitable reference genes for qRT- PCR studies in *M. officinalis* or lemon balm. We ranked ten commonly used reference genes based on their expression stabilities. Based on the results, we recommend using a combination of *EF-1α* and *GAPDH* for the normalization of qRT-PCR in lemon balm. *HLH71* was identified as the least stable gene. The identified optimal and the least stable genes were then validated with the *PAL* gene under elicitor stress. In conclusion, this study will provide a useful resource for more accurate and widespread experiments with qRT-PCR in *M. officinalis* and other related species.

## Figures and Tables

**Table 1:** Primer information of the candidate reference genes.

Gene Name	Primer Sequence (5′-3′)		Annealing Temperature	Amplicon Size (bp)	Primer Efficiency	R^2^ Value
	Forward Primer	Reverse Primer				
*60S rRNA*	TTGTCGGGAAATATGGCACC	CCTTCACTTTGCCACAGTCC	59.1 °C	175	101.73%	1.00
*28S rRNA*	CAACTGGTCAAAGATCCGCC	CCTTCTGACGACGCTCTAAC	59.1 °C	170	104.06%	0.99
*Ubiquitin*	GAGCTCTCCACCTCCAAAGT	ACGCACTCTGGTTGACTACA	56.8 °C	152	100.87%	0.99
*ATP synthase*	GGCTTGAACGAAACGGAAGA	AGAGTTGGTTTGACTGCCCT	59.5 °C	150	101.35%	0.91
*F1-ATPase*	TATCTGTCAGTCGTGTCGGG	AAAGGCGGCTACTTCTCGAT	59.1 °C	150	101.14%	0.99
*GAPDH*	CCACTCCACTTTCGCTCTCT	TGAAGGGATTGTTGACAGCA	59.5 °C	181	97.00%	1.00
*EF-1α*	TGACAACGAAACGCAACACA	CATTGGGTACTTGACAGGCG	59.5 °C	180	97.33%	1.00
*β-tubulin*	AAAGGTCACTACACGGAGGG	GAGATCAGCAGGGTTCCCAT	58.4 °C	181	100.08%	1.00
*HLH71*	CGAACGAAACCGCCGAAA	TGGCTTCTAGTGACTGCAGA	59.1 °C	181	97.61%	0.97
*GST*	TGGCCAGCACTCCTTTATCA	CTTGCATTAGGGTGGATGGC	61 °C	180	104.86%	1.00

**Table 2:** Expression stability of candidate genes analyzed by ΔCt and BestKeeper.

Rank	Plant Organs				Heat Stress			
	Genes	Average of STDEV	Genes	Std dev [+/− CP]	Genes	Average of STDEV	Genes	Std dev [+/− CP]
1	*GAPDH*	1.78	*GST*	1.22	*28S rRNA*	1.04	*HLH71*	0.44
2	*F1-ATPase*	1.91	*28S rRNA*	1.41	*Ubiquitin*	1.06	*GST*	1.5
3	*28S rRNA*	1.92	*HLH71*	1.95	*EF-1α*	1.07	*F1-ATPase*	1.53
4	*β-tubulin*	1.94	*ATP-synthase*	2.52	*β-tubulin*	1.08	*β-tubulin*	2.08
5	*60SrRNA*	2.01	*β-tubulin*	2.58	*GAPDH*	1.14	*EF-1α*	2.33
6	*EF-1α*	2.01	*F1-ATPase*	2.62	*GST*	1.38	*28S rRNA*	2.41
7	*GST*	2.01	*GAPDH*	2.64	*60S rRNA*	1.65	*Ubiquitin*	2.6
8	*ATP-synthase*	2.12	*60S rRNA*	2.78	*ATP-synthase*	1.73	*GAPDH*	2.82
9	*Ubiquitin*	3.11	*EF-1α*	2.98	*F1-ATPase*	1.83	*60S rRNA*	3.25
10	*HLH71*	3.19	*Ubiquitin*	3.65	*HLH71*	2.5	*ATP-synthase*	3.57
**Rank**	**Cold stress**				**Salt stress**			
	**Genes**	**Average of STDEV**	**Genes**	**Std dev [+/− CP]**	**Genes**	**Average of STDEV**	**Genes**	**Std dev [+/− CP]**
1	*EF-1α*	0.92	*60S rRNA*	0.72	*GAPDH*	0.83	*GST*	0.79
2	*GST*	0.95	*F1-ATPase*	0.86	*β-tubulin*	0.85	*F1-ATPase*	1.57
3	*ATP-synthase*	0.95	*28S rRNA*	1.34	*28S rRNA*	0.89	*60S rRNA*	1.82
4	*GAPDH*	0.99	*GST*	1.54	*EF-1α*	0.9	*28S rRNA*	2.02
5	*28S rRNA*	1.00	*ATP-synthase*	1.63	*HLH71*	1.11	*GAPDH*	2.27
6	*β-tubulin*	1.31	*EF-1α*	1.91	*F1-ATPase*	1.29	*β-tubulin*	2.31
7	*HLH71*	1.42	*GAPDH*	2.15	*60S rRNA*	1.29	*HLH71*	2.43
8	*Ubiquitin*	1.49	*HLH71*	2.26	*Ubiquitin*	1.39	*EF-1α*	2.47
9	*F1-ATPase*	1.54	*β-tubulin*	2.44	*ATP-synthase*	1.43	*Ubiquitin*	3.21
10	*60S rRNA*	1.58	*Ubiquitin*	2.72	*GST*	1.8	*ATP-synthase*	3.28
**Rank**	**Osmotic stress**				**All stress together**			
	**Genes**	**Average of STDEV**	**Genes**	**Std dev [+/− CP]**	**Genes**	**Average of STDEV**	**Genes**	**Std dev [+/− CP]**
1	*28S rRNA*	2.26	*HLH71*	0.48	*28S rRNA*	1.93	*GST*	1.19
2	*60S rRNA*	2.27	*F1-ATPase*	0.76	*EF-1α*	2.01	*F1-ATPase*	1.29
3	*β-tubulin*	2.28	*GST*	0.87	*GAPDH*	2.03	*28S rRNA*	1.42
4	*EF-1α*	2.43	*28S rRNA*	2.02	*β-tubulin*	2.07	*β-tubulin*	1.44
5	*GAPDH*	2.49	*β-tubulin*	2.19	*Ubiquitin*	2.15	*EF-1α*	2.02
6	*Ubiquitin*	2.59	*60S rRNA*	3.62	*F1-ATPase*	2.47	*GAPDH*	2.1
7	*F1-ATPase*	3.06	*EF-1α*	4.3	*60S rRNA*	2.48	*HLH71*	2.11
8	*GST*	3.11	*GAPDH*	4.44	*GST*	2.51	*Ubiquitin*	2.24
9	*HLH71*	3.39	*Ubiquitin*	4.61	*ATP-synthase*	3.24	*60S rRNA*	2.7
10	*ATP-synthase*	4.31	*ATP-synthase*	6.54	*HLH71*	3.26	*ATP-synthase*	3.5
**Rank**	**In vitro organs**				**All conditions combined**			
	**Genes**	**Average of STDEV**	**Genes**	**Std dev [+/− CP]**	**Genes**	**Average of STDEV**	**Genes**	**Std dev [+/− CP]**
1	*β-tubulin*	1.21	*HLH71*	1.87	*28SrRNA*	2.04	*F1-ATPase*	0.62
2	*Ubiquitin*	1.23	*F1-ATPase*	2.35	*GAPDH*	2.13	*GST*	0.74
3	*GAPDH*	1.26	*GST*	2.67	*β-tubulin*	2.15	*β-tubulin*	0.86
4	*ATP-synthase*	1.34	*28S rRNA*	2.71	*EF-1α*	2.28	*28S rRNA*	1.07
5	*GST*	1.36	*β-tubulin*	3.2	*GST*	2.32	*EF-1α*	1.21
6	*28S rRNA*	1.49	*Ubiquitin*	3.35	*60S rRNA*	2.55	*HLH71*	1.27
7	*F1-ATPase*	1.58	*GAPDH*	3.38	*F1-ATPase*	2.58	*Ubiquitin*	1.55
8	*HLH71*	1.78	*ATP-synthase*	3.71	*ATP-synthase*	2.91	*GAPDH*	1.56
9	*EF-1α*	2.27	*60S rRNA*	3.94	*Ubiquitin*	2.98	*60S rRNA*	1.72
10	*60S rRNA*	2.4	*EF-1α*	4.91	*HLH71*	3.34	*ATP-synthase*	2.32

**Table 3:** Expression stability of candidate genes analyzed by NormFinder.

Rank	Plant Organs		Heat Stress	
	Gene Name	Stability Value	Gene Name	Stability Value
1	*GAPDH*	0.16	*GAPDH*	0.10
2	*ATP-synthase*	0.17	*β-tubulin*	0.14
3	*β-tubulin*	0.20	*28S rRNA*	0.17
4	*GST*	0.21	*ATP-synthase*	0.18
5	*Ubiquitin*	0.22	*EF-1α*	0.18
6	*HLH71*	0.23	*HLH71*	0.23
7	*60S rRNA*	0.30	*Ubiquitin*	0.24
8	*EF-1α*	0.30	*GST*	0.36
9	*F1-ATPase*	0.47	*F1-ATPase*	0.46
10	*28S rRNA*	0.49	*60S rRNA*	0.47
**Best combination**	*GAPDH* and *β-tubulin*	0.12	*GAPDH* and *β-tubulin*	0.06
**Rank**	**Cold stress**		**Salt stress**	
	**Gene name**	**Stability value**	**Gene name**	**Stability value**
1	*GAPDH*	0.120	*GAPDH*	0.09
2	*28S rRNA*	0.188	*28S rRNA*	0.11
3	*EF-1α*	0.191	*EF-1α*	0.14
4	*Ubiquitin*	0.209	*β-tubulin*	0.14
5	*F1-ATPase*	0.269	*ATP-synthase*	0.18
6	*ATP-synthase*	0.286	*GST*	0.24
7	*GST*	0.333	*HLH71*	0.26
8	*60S rRNA*	0.358	*F1-ATPase*	0.29
9	*β-tubulin*	0.485	*Ubiquitin*	0.33
10	*HLH71*	1.015	*60S rRNA*	0.33
**Best combination**	*GAPDH and F1-ATPase*	0.125	*28S rRNA and β-tubulin*	0.07
**Rank**	**Osmotic stress**		**All stress together**	
	**Gene name**	**Stability value**	**Gene name**	**Stability value**
1	*28S rRNA*	0.11	*GAPDH*	0.17
2	*ATP-synthase*	0.11	*28S rRNA*	0.18
3	*Ubiquitin*	0.12	*ATP-synthase*	0.24
4	*GAPDH*	0.16	*EF-1α*	0.28
5	*HLH71*	0.18	*β-tubulin*	0.32
6	*EF-1α*	0.19	*Ubiquitin*	0.39
7	*β-tubulin*	0.20	*GST*	0.46
8	*60S rRNA*	0.22	*F1-ATPase*	0.49
9	*F1-ATPase*	0.26	*60S rRNA*	0.54
10	*GST*	0.27	*HLH71*	0.66
**Best combination**	*ATP-synthase* and *28S rRNA*	0.07	*GAPDH* and *28S rRNA*	0.13
**Rank**	**In vitro organs**		**All conditions combined**	
	**Gene name**	**Stability value**	**Gene name**	**Stability value**
1	*EF-1α*	0.12	*GAPDH*	0.25
2	*β-tubulin*	0.22	*ATP-synthase*	0.29
3	*GST*	0.24	*EF-1α*	0.29
4	*HLH71*	0.40	*β-tubulin*	0.34
5	*GAPDH*	0.49	*Ubiquitin*	0.43
6	*ATP-synthase*	0.52	*28S rRNA*	0.45
7	*Ubiquitin*	0.62	*GST*	0.46
8	*F1-ATPase*	0.66	*HLH71*	0.53
9	*28S rRNA*	0.76	*F1-ATPase*	0.60
10	*60S rRNA*	2.47	*60S rRNA*	0.86
**Best combination**	*EF-1α* and *β-tubulin*	0.14	*EF-1α* and *GAPDH*	0.22

**Table 4:** Comprehensive ranking of candidate genes analyzed by RefFinder.

	Plant Organs		Heat Stress	
Rank	Genes	Geomean of Ranking Values	Genes	Geomean of Ranking Values
*1*	*GAPDH*	1.93	*28S rRNA*	1.86
*2*	*28S rRNA*	2.45	*EF-1α*	2.59
*3*	*GST*	3.48	*Ubiquitin*	2.74
*4*	*F1-ATPase*	3.94	*β-tubulin*	3.94
*5*	*EF-1α*	4.41	*GST*	4.9
*6*	*β-tubulin*	4.68	*GAPDH*	5.32
*7*	*60S rRNA*	5.57	*HLH71*	5.62
*8*	*ATP-synthase*	5.98	*F1-ATPase*	6.64
*9*	*HLH71*	7.4	*60SrRNA*	7.45
*10*	*Ubiquitin*	9.24	*ATP-synthase*	8.11
	**Cold stress**		**Salt stress**	
**Rank**	**Genes**	**Geomean of ranking values**	**Genes**	**Geomean of ranking values**
*1*	*GST*	1.68	*GAPDH*	1.5
*2*	*EF-1α*	2.63	*β-tubulin*	2.21
*3*	*28S rRNA*	2.78	*28S rRNA*	3.46
*4*	*ATP-synthase*	3.41	*EF-1α*	4.43
*5*	*GAPDH*	5.14	*F1-ATPase*	5.05
*6*	*60S rRNA*	5.62	*HLH71*	5.44
*7*	*F1-ATPase*	6.18	*GST*	5.62
*8*	*β-tubulin*	6.64	*60S rRNA*	5.86
*9*	*HLH71*	7.48	*Ubiquitin*	7.67
*10*	*Ubiquitin*	8.18	*ATP-synthase*	8.68
	**Osmotic stress**		**All stress together**	
**Rank**	**Genes**	**Geomean of ranking values**	**Genes**	**Geomean of ranking values**
*1*	*28S rRNA*	2.91	*28S rRNA*	1.86
*2*	*β-tubulin*	2.94	*EF-1α*	2.34
*3*	*60S rRNA*	3.13	*GAPDH*	2.91
*4*	*EF-1α*	3.25	*β-tubulin*	3.56
*5*	*GAPDH*	3.76	*GST*	4.76
*6*	*F1-ATPase*	5.12	*F1-ATPase*	4.92
*7*	*HLH71*	5.2	*Ubiquitin*	4.95
*8*	*Ubiquitin*	5.58	*60S rRNA*	6.9
*9*	*GST*	6.26	*HLH71*	8.91
*10*	*ATP-synthase*	10	*ATP-synthase*	9.49
	**In vitro organs**		**All conditions combined**	
**Rank**	**Genes**	**Geomean of ranking values**	**Genes**	**Geomean of ranking values**
*1*	*β-tubulin*	2.11	*28S rRNA*	1.86
*2*	*Ubiquitin*	2.21	*EF-1α*	2.11
*3*	*ATP-synthase*	3.56	*β-tubulin*	3.22
*4*	*GAPDH*	3.71	*GAPDH*	3.36
*5*	*GST*	4.16	*GST*	4.33
*6*	*HLH71*	4.76	*F1-ATPase*	4.6
*7*	*F1-ATPase*	5.12	*60S rRNA*	6.64
*8*	*28S rRNA*	5.42	*Ubiquitin*	6.65
*9*	*EF-1α*	9.24	*HLH71*	8.8
*10*	*60S rRNA*	9.74	*ATP-synthase*	9.24

## Data Availability

Data is contained within the article.

## References

[1] Shakeri A., Sahebkar A., Javadi B. (2016). *Melissa officinalis* L.—A review of its traditional uses, phytochemistry and pharmacology. J. Ethnopharmacol..

[2] Draginic N., Jakovljevic V., Andjic M., Jeremic J., Srejovic I., Rankovic M., Tomovic M., Nikolic Turnic T., Svistunov A., Bolevich S. (2021). *Melissa officinalis* L. as a nutritional strategy for cardioprotection. Front. Physiol..

[3] Mahboubi M., Taghizadeh M., Talaei S.A., Takht Firozeh S.M., Rashidi A.A., Tamtaji O.R. (2016). Combined administration of *Melissa officinalis* and *Boswellia serrata* extracts in an animal model of memory. Iran. J. Psychiatry Behav. Sci..

[4] Heshmati J., Morvaridzadeh M., Sepidarkish M., Fazelian S., Rahimlou M., Omidi A., Palmowski A., Asadi A., Shidfar F. (2020). Effects of *Melissa officinalis* (Lemon Balm) on cardio-metabolic outcomes: A systematic review and meta-analysis. Phytother. Res..

[5] Božović M., Garzoli S., Baldisserotto A., Romagnoli C., Pepi F., Cesa S., Vertuani S., Manfredini S., Ragno R. (2018). *Melissa officinalis* L. subsp. altissima (Sibth. & Sm.) Arcang. Essential Oil: Chemical composition and preliminary antimicrobial investigation of samples obtained at different harvesting periods and by fractionated extractions. Ind. Crops Prod..

[6] Kianersi F., Amin Azarm D., Pour-Aboughadareh A., Poczai P. (2022). Change in secondary metabolites and expression pattern of key rosmarinic acid related genes in iranian lemon balm (*Melissa officinalis* L.) ecotypes using methyl jasmonate treatments. Molecules.

[7] Tonelli M., Pellegrini E., D’Angiolillo F., Petersen M., Nali C., Pistelli L., Lorenzini G. (2015). Ozone-elicited secondary metabolites in shoot cultures of *Melissa officinalis* L. Plant Cell Tissue Organ Cult..

[8] Ramawat K.G., Ramawat K.G., Ekiert H.M., Goyal S. (2021). An introduction to the process of cell, tissue, and organ differentiation, and production of secondary metabolites. Plant Cell and Tissue Differentiation and Secondary Metabolites.

[9] Bhaskar R., Xavier L.S.E., Udayakumaran G., Kumar D.S., Venkatesh R., Nagella P. (2022). Biotic elicitors: A boon for the in-vitro production of plant secondary metabolites. Plant Cell Tissue Organ Cult..

[10] Fooladi vanda G., Shabani L., Razavizadeh R. (2019). Chitosan enhances rosmarinic acid production in shoot cultures of *Melissa officinalis* L. through the induction of methyl jasmonate. Bot. Stud..

[11] Mansouri M., Mohammadi F. (2021). Transcriptome analysis to identify key genes involved in terpenoid and rosmarinic acid biosynthesis in lemon balm (*Melissa officinalis*). Gene.

[12] Sen M.K., Hamouzová K., Košnarová P., Roy A., Soukup J. (2021). Identification of the most suitable reference gene for gene expression studies with development and abiotic stress response in *Bromus sterilis*. Sci. Rep..

[13] Chen M., Wang B., Li Y., Zeng M., Liu J., Ye X., Zhu H., Wen Q. (2021). Reference gene selection for QRT-PCR Analyses of luffa (*Luffa cylindrica*) plants under abiotic stress conditions. Sci. Rep..

[14] Oneto C.D., Bossio E., Faccio P., Beznec A., Lewi D. (2017). Validation of housekeeping genes for QPCR in maize during water deficit stress conditions at flowering time. Maydica.

[15] Galli V., Borowski J.M., Perin E.C., Messias R.d.S., Labonde J., Pereira I.d.S., Silva S.D.d.A., Rombaldi C.V. (2015). Validation of reference genes for accurate normalization of gene expression for real time-quantitative PCR in strawberry fruits using different cultivars and osmotic stresses. Gene.

[16] Joseph J.T., Poolakkalody N.J., Shah J.M. (2018). Plant reference genes for development and stress response studies. J. Biosci..

[17] Czechowski T., Stitt M., Altmann T., Udvardi M.K., Scheible W.-R. (2005). Genome-wide identification and testing of superior reference genes for transcript normalization in arabidopsis. Plant Physiol..

[18] Dong X.-M., Zhang W., Zhang S.-B. (2022). Selection and validation of reference genes for quantitative real-time PCR analysis of development and tissue-dependent flower color formation in *Cymbidium lowianum*. Int. J. Mol. Sci..

[19] Yin H., Yin D., Zhang M., Gao Z., Tuluhong M., Li X., Li J., Li B., Cui G. (2022). Validation of appropriate reference genes for QRT–PCR normalization in oat (*Avena sativa* L.) under UV-B and high-light stresses. Int. J. Mol. Sci..

[20] Aminfar Z., Rabiei B., Tohidfar M., Mirjalili M.H. (2019). Selection and validation of reference genes for quantitative real-time PCR in *Rosmarinus officinalis* L. in various tissues and under elicitation. Biocatal. Agric. Biotechnol..

[21] Pfaffl M.W., Tichopad A., Prgomet C., Neuvians T.P. (2004). Determination of stable housekeeping genes, differentially regulated target genes and sample integrity: BestKeeper–Excel-based tool using pair-wise correlations. Biotechnol. Lett..

[22] Silver N., Best S., Jiang J., Thein S.L. (2006). Selection of housekeeping genes for gene expression studies in human reticulocytes using real-time PCR. BMC Mol. Biol..

[23] Vandesompele J., De Preter K., Pattyn F., Poppe B., Van Roy N., De Paepe A., Speleman F. (2002). Accurate normalization of real-time quantitative RT-PCR data by geometric averaging of multiple internal control genes. Genome Biol..

[24] Andersen C.L., Jensen J.L., Ørntoft T.F. (2004). Normalization of real-time quantitative reverse transcription-PCR data: A model-based variance estimation approach to identify genes suited for normalization, applied to bladder and colon cancer data sets. Cancer Res..

[25] Xie F., Xiao P., Chen D., Xu L., Zhang B. (2012). MiRDeepFinder: A MiRNA analysis tool for deep sequencing of plant small RNAs. Plant Mol. Biol..

[26] El-Naggar H.M., Read P.E. (2010). PAL Gene activity and rosmarinic acid production in rosemary genotypes. J. Herbs Spices Med. Plants.

[27] Vyas P., Mukhopadhyay K. (2018). Elicitation of phenylpropanoids and expression analysis of PAL gene in suspension cell culture of *Ocimum tenuiflorum* L. Proc. Natl. Acad. Sci. India Sect. B Biol. Sci..

[28] Kahila M.M.H., Najy A.M., Rahaie M., Mir-Derikvand M. (2018). Effect of nanoparticle treatment on expression of a key gene involved in thymoquinone biosynthetic pathway in *Nigella Sativa* L. Nat. Prod. Res..

[29] Jalali S., Salami S.A., Sharifi M., Sohrabi S. (2019). Signaling compounds elicit expression of key genes in cannabinoid pathway and related metabolites in cannabis. Ind. Crops Prod..

[30] Devi K., Mishra S.K., Sahu J., Panda D., Modi M.K., Sen P. (2016). Genome wide transcriptome profiling reveals differential gene expression in secondary metabolite pathway of *Cymbopogon winterianus*. Sci. Rep..

[31] Aminfar Z., Rabiei B., Tohidfar M., Mirjalili M.H. (2019). Identification of key genes involved in the biosynthesis of triterpenic acids in the mint family. Scietific Rep..

[32] Bolhassani M., Niazi A., Tahmasebi A., Moghadam A. (2021). Identification of key genes associated with secondary metabolites biosynthesis by system network analysis in *Valeriana officinalis*. J. Plant Res..

[33] Abdollahi Mandoulakani B., Eyvazpour E., Ghadimzadeh M. (2017). The effect of drought stress on the expression of key genes involved in the biosynthesis of phenylpropanoids and essential oil components in basil (*Ocimum Basilicum* L.). Phytochemistry.

[34] Negrutskii B.S., El’skaya A.V. (1998). Eukaryotic translation elongation factor 1α: Structure, expression, functions, and possible role in aminoacyl-TRNA channeling. Progress in Nucleic Acid Research and Molecular Biology.

[35] Tristan C., Shahani N., Sedlak T.W., Sawa A. (2011). The diverse functions of GAPDH: Views from different subcellular compartments. Cell. Signal..

[36] Ashrafi M., Azimi Moqadam M.R., Moradi P., Mohsenifard E., Shekari F. (2018). Evaluation and validation of housekeeping genes in two contrast species of thyme plant to drought stress using real-time PCR. Plant Physiol. Biochem..

[37] Gopalam R., Rupwate S.D., Tumaney A.W. (2017). Selection and validation of appropriate reference genes for quantitative real-time PCR analysis in *Salvia hispanica*. PLoS ONE.

[38] Lian C., Zhang B., Yang J., Lan J., Yang H., Guo K., Li J., Chen S. (2022). Validation of suitable reference genes by various algorithms for gene expression analysis in isodon rubescens under different abiotic stresses. Sci. Rep..

[39] Yang Y., Hou S., Cui G., Chen S., Wei J., Huang L. (2010). Characterization of reference genes for quantitative real-time PCR analysis in various tissues of *Salvia miltiorrhiza*. Mol. Biol. Rep..

[40] Borah B., Hussain M., Wann S.B., Bhau B.S. (2020). Selection and Validation of suitable reference genes for quantitative real time PCR analysis of gene expression studies in patchouli under meloidogyne incognita attack and PGPR treatment. Gene Rep..

[41] Bharati R., Sen M.K., Kumar R., Gupta A., Sur V.P., Melnikovová I., Fernández-Cusimamani E. (2022). Selection and validation of the most suitable reference genes for quantitative real-time PCR normalization in *Salvia rosmarinus* under in vitro conditions. Plants.

[42] Wang H.-L., Chen J., Tian Q., Wang S., Xia X., Yin W. (2014). Identification and validation of reference genes for populus euphratica gene expression analysis during abiotic stresses by quantitative real-time PCR. Physiol. Plant..

[43] Valenzuela F., D’Afonseca V., Hernández R., Gómez A., Arencibia A.D. (2022). Validation of reference genes in a population of blueberry (*Vaccinium corymbosum*) plants regenerated in colchicine. Plants.

[44] Beranová K., Bharati R., Žiarovská J., Bilčíková J., Hamouzová K., Klíma M., Fernández-Cusimamani E. (2022). Morphological, cytological, and molecular comparison between diploid and induced autotetraploids of *Callisia fragrans* (Lindl.) woodson. Agronomy.

